# End‐of‐Life Care Experiences, Attitudes and Perceptions of Intensive Care Clinicians in Middle Eastern Countries: A Systematic Integrative Review

**DOI:** 10.1111/nicc.70201

**Published:** 2025-10-05

**Authors:** Khalidah Mobarki, Ping Guo, Misbah Ismail Mobarki, Nikolaos Efstathiou

**Affiliations:** ^1^ College of Medicine and Health, School of Health Sciences, Department of Nursing and Midwifery University of Birmingham Birmingham UK; ^2^ Nursing College Jazan University Jazan Saudi Arabia; ^3^ Samtah General Hospital Jazan Saudi Arabia

**Keywords:** end‐of‐life care, experiences, intensive care units, Middle Eastern countries, systematic review

## Abstract

**Background:**

End‐of‐life care (EoLC) in Intensive Care Units (ICUs) in Middle Eastern (ME) countries is influenced by cultural and legal constraints, creating unique ethical dilemmas and challenges for clinicians. Ethical debates surround the acceptability of withholding and withdrawing treatments that sustain patients' lives.

**Aim:**

To collate and synthesise existing evidence on the EoLC experiences, attitudes and perspectives of ICU clinicians in Middle Eastern countries.

**Study Design:**

A systematic integrative review was conducted. Searches were completed in AMED, CINAHL, EMBASE, Medline, PubMed and Google Scholar for studies published between 2012 and 2024. Quantitative results were translated into textual data and, along with the qualitative findings, were analysed using the thematic synthesis approach.

**Results:**

Twenty‐seven studies met the inclusion criteria: 16 quantitative, 10 qualitative and one mixed methods. Five themes were developed: (1) Challenges in EoLC decision‐making in ICUs; (2) Cultural and ethical issues impacting the delivery of EoLC; (3) The need for comprehensive EoLC guidelines; (4) EoLC education and training for ICU clinicians; and (5) holistic support systems for ICU clinicians.

**Conclusions:**

Challenges in providing EoLC in Middle Eastern ICUs concern communication, problematic interactions with families and decision‐making. Cultural and ethical issues are also found to affect EoLC delivery, pointing to the importance of comprehensive and clear guidelines. Future research could explore EoLC from the perspective of a wider range of stakeholders in Middle Eastern countries and determine how international best practices can be adjusted to Middle Eastern ICUs to enhance EoLC provision.

**Relevance to Clinical Practice:**

To enhance EoLC in Middle Eastern ICUs, clinicians need to undergo training on communication skills, cultural sensitivity and family involvement strategies. Organisational support is required to better guide communication with families and palliative care decisions.

**Impact Statements**

- What is known about the topic
  ○ EoLC in Intensive Care Units is ethically and emotionally complex, particularly in the Middle East, where cultural and religious factors play a significant role in decision‐making.
  ○ ICU clinicians often face challenges when deciding on life‐sustaining treatments, balancing family wishes, legal constraints and professional ethics.
  ○ There is a lack of standardised EoLC guidelines across Middle Eastern ICUs and regional variations in attitudes towards EoLC contribute to inconsistent care delivery.
- What this paper adds
  ○ This systematic integrative review identifies key themes that affect EoLC in Middle Eastern ICUs, including challenges in decision‐making, cultural and ethical issues, the need for comprehensive guidelines and the importance of EoLC education.
  ○ It emphasises the need for structured communication training, cultural sensitivity and enhanced family involvement strategies to improve EoLC practices in Middle Eastern ICUs.

## 1 Introduction

In intensive care units (ICUs), multidisciplinary teams provide intensive and specialised medical and nursing care to individuals with life‐threatening conditions [1]. Due to technological improvements, modern ICUs offer more effective and efficient care; however, mortality rates in ICUs are still high, varying from 16.2% to 46.8% across the globe [2, 3, 4, 5]. More than 70% of ICU deaths happen after care providers limit or withdraw life‐sustaining treatments, [6] making end‐of‐life care (EoLC) a common aspect of intensive care provision.

The transition from maintaining life support treatments to EoLC for ICU patients is often associated with conflicts, misunderstandings and moral and professional dilemmas [6]. Ethical debates surround the acceptability of withholding and withdrawing treatments that sustain patients' lives. Although most ethicists agree that withholding and withdrawing treatment are equivalent, [7] some scholars and practitioners disagree on whether and how physicians can be involved in withholding and withdrawing treatments [8]. Healthcare providers may also want to avoid the delivery of futile treatment, defined as ‘interventions that cannot accomplish the intended physiological goals’ because it undermines patient dignity [9].

The doctrine of double effect (a philosophical principle that justifies doing a ‘good action’ with a potentially ‘bad effect’) makes it difficult for care providers to decide on activities that can potentially lead to both positive and negative consequences for patients [10]. For instance, care provided to dying patients to alleviate pain, restlessness and agitation during terminal care may be ethically ambiguous as it intends to decrease pain and suffering but may shorten life [11]. It may also be challenging for clinicians to balance paternalism as the care philosophy with the expectations of shared decision‐making and respect for patient autonomy. Legal challenges surrounding the provision of care to patients who lack decision‐making capacity result in medical professionals being afraid to take responsibility for care decisions, which contradicts the paternalistic philosophy of care. At the same time, the inclusion of family members in decision‐making may further complicate the work of nurses and physicians in ICUs as these people may not have a good understanding of EoLC and thus may not be able to make informed decisions in patients' best interests [11].

Cultural and religious expectations and beliefs are additional factors to consider when providing EoLC in ICUs. In Middle Eastern countries (typically including the Arabian Peninsula, the Levant, Iraq, Iran, Turkey and Egypt), dominating religions such as Islam and Judaism inform healthcare laws, policies and practices; these religions emphasise the sanctity of life and human dignity, which is reflected in laws that prohibit withdrawing life‐sustaining treatments [12]. Moral distress can develop in such situations when religious and moral obligations directly contradict professional obligations and responsibilities [12, 13]. The very nature of EoLC decision‐making in this region may differ from the conventional process adopted in the West, which involves decisions about resuscitation, advance care planning, palliative care or the preferred setting for dying [14]. Religious and cultural norms play a big role in clinicians', patients', and family members' understanding of what is appropriate in EoLC decision‐making; as a result, non‐beneficial treatment may be chosen that aligns with religious beliefs but not with the patient's needs and wishes [12].

Many researchers have sought solutions to the cultural and religious dilemmas faced by ICU practitioners in the Middle East by exploring how to simultaneously respect religious values while also attending to the unique needs of ICU patients and their families [12, 13, 15]. Discrepancies in the interpretations and practices of religions among patients and healthcare providers (many of whom come from non‐ME countries) create conflicting situations related to end‐of‐life care. To the authors' best knowledge, no attempt has been made to collate and synthesise studies on ICU healthcare providers' EoLC experiences, attitudes and perspectives in the Middle East. Due to significant legal restrictions on the EoLC practice in this region, as well as unique cultural and religious beliefs that dictate EoLC, it is important to undertake a review focusing on these specific issues within the Middle East [16]. Synthesised findings could be used for developing relevant training and support programmes for ICU nurses and doctors.

## 2 Aim

This systematic integrative review aimed to collect and synthesise existing evidence on ICU healthcare providers' experiences, attitudes and perspectives regarding the delivery of EoLC in ICUs within Middle Eastern countries.

## 3 Design and Methods

### 3.1 Search Strategy

A list of relevant keywords was formulated with the help of the P (Population), I (phenomena of Interest) and Co (the context) (PICo) strategy [17]. In addition, we used Medical Subject Heading (MeSH) terms, relevant terminology and truncation symbols (*) to enhance our search strategy (Table 1) [18]. Boolean operators (AND, OR) were also applied to connect keywords in a search, thus achieving a greater number of more focused search results [19]. A comprehensive search was conducted across five databases (MEDLINE, PubMed, EMBASE, CINAHL and AMED), supplemented by searches in the academic search engine Google Scholar, using the identified keywords. The final database searches were conducted on 12 November 2024. A subject‐specific librarian reviewed and validated the search strategy, which is presented in Appendix A: Table A1.

**TABLE 1:** Inclusion and exclusion criteria.

Criteria category	Inclusion criteria	Exclusion criteria
Focus	Studies focusing on healthcare providers' experiences, attitudes and perceptions related to EoLC within ICUs or Do Not Resuscitate (DNR) cases.	Studies not primarily focused on end‐of‐life or DNR patients, including those on general palliative care or involving general staff.
Population/setting	Doctors, nurses exclusively working in Middle Eastern ICUs.	Healthcare providers from non‐Middle Eastern or non‐ICU settings, such as acute care, hospice or home care.
Methodology	Quantitative, qualitative or mixed methods studies.	Study protocols, expert commentaries, opinions, editorials, letters to the editor, conference abstracts, dissertations and literature reviews.
Publication date	Studies published from 1 January 2012 to October 2024.	Studies published before January 2012 or after October 2024.
Publication type	Peer‐reviewed empirical studies published in scientific journals.	Non‐peer‐reviewed publications. Grey literature.
Language	Publications in English or Arabic.	Studies published in languages other than English or Arabic.
Accessibility	Studies with full‐text access for thorough review and analysis.	Unpublished or inaccessible studies.

### 3.2 Inclusion and Exclusion Criteria

Table 1 contains a list of the inclusion and exclusion criteria used to select relevant studies. Studies published earlier than January 2012 were excluded because they were not likely to reflect the current state of ICU practice in the Middle East.

### 3.3 Study Selection

**FIGURE 1:**
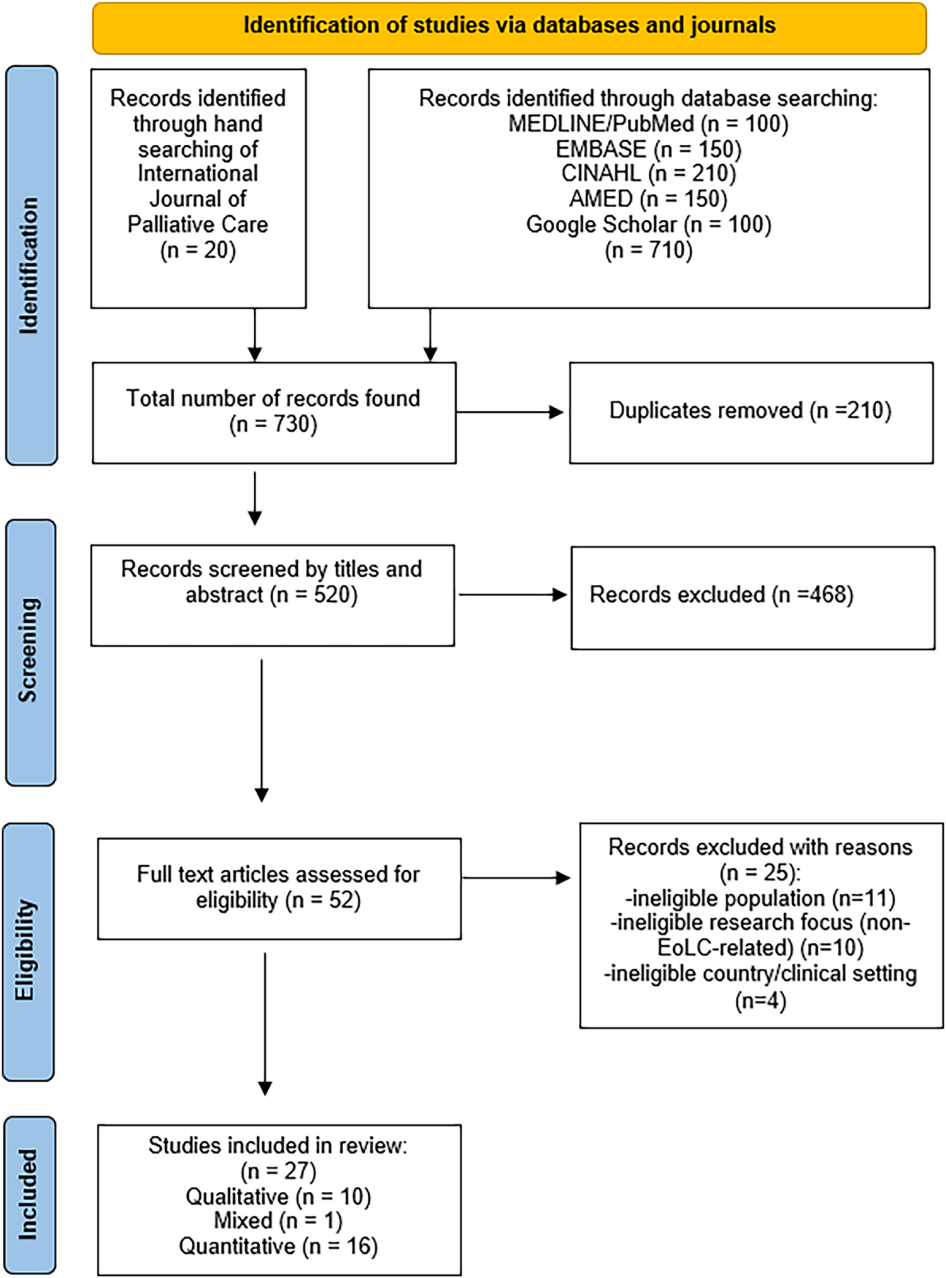
PRISMA chart.

The retrieved studies were exported from each database into EndNote^tm^ version 20 to check for duplicates. However, since EndNote's sensitivity for deduplication does not exceed 57%, manual deduplication was also performed to ensure that only unique studies were included [20]. The next step involved uploading the studies into Rayyan, an online platform designed to support reviewers conducting systematic reviews [21, 22]. Two reviewers (K.M., M.M.) then screened the titles, abstracts and reference lists of the studies identified as eligible. During the screening of reference lists of eligible studies, it was identified that some relevant studies from the International Journal of Palliative Care Nursing had not been retrieved via the database searches; hence, the contents of this journal were also searched. After the title and abstract screening, two reviewers (K.M., N.E.) independently screened the full texts of all potentially relevant studies. The final list of studies to be included in the integrative review was collectively determined and approved by the reviewers (K.M., N.E.). A PRISMA flowchart documenting the results of the search and how the sample was narrowed down to 27 studies is provided in Figure 1.

### 3.4 Data Collection Tools and Methods

The quality of the qualitative, [23] cross‐sectional, [24] cohort, [25] and case–control studies [26] was appraised using the Joanna Briggs Institute (JBI) Critical Appraisal Tools [27]. The Mixed Methods Appraisal Tool (MMAT) was employed for mixed methods studies [28]. Two reviewers (K.M., M.M.) independently appraised the included studies, and a high inter‐rater agreement of 95% was achieved. The studies were categorised into ‘Good’ or ‘Fair’ groups as per the JBI [27] and MMAT [28] criteria. Studies included in the ‘Good’ category had a high methodological rigour compared to the ‘Fair’ studies that had some minor methodological flaws (see Appendix B). No studies were excluded based on quality.

### 3.5 Analysis and Synthesis of Literature

The JBI [29] approach was used to integrate quantitative data qualitatively, that is, to translate numerical findings into text. According to JBI, this process involves extracting data from quantitative studies and translating or converting it into ‘textual descriptions’ to allow integration with qualitative data [29]. Following this, thematic synthesis of data, as described by Thomas and Harden, [21] was undertaken. It consisted of three key steps: the coding of textual data ‘line‐by‐line’, the formation of descriptive themes and the development of analytical themes [21].

## 4 Results

The comprehensive search across the selected databases generated 710 records. An additional 20 publications were accessed using manual searches of the International Journal of Palliative Care Nursing. Automated and manual deduplication removed 210 duplicates, leaving 520 unique records for further examination. Manual screening of these studies' titles and abstracts excluded 468 studies. Of these, 367 were excluded because of the ineligible focus on non‐EoLC‐related contexts, 67 were excluded because they were set in a non‐ICU setting, and the remaining 34 were ineligible because of the population or country. The full text of the remaining 52 publications was scanned to determine their eligibility. Ultimately, 27 studies met the inclusion criteria and were selected for analysis and synthesis.

The 27 studies included in this review were conducted in Saudi Arabia (*n* = 7), Iran (*n* = 6), Jordan (*n* = 5), Egypt (*n* = 4), Israel (*n* = 3), Bahrain (*n* = 1) and Palestine (*n* = 1). They represent a diversity of methodological approaches: ten qualitative studies, 16 quantitative studies and one mixed‐methods study. Across the 27 studies, 2387 participants were enrolled. Sample sizes differed depending on the methodological approach; for example, qualitative studies included as few as five participants [22], and quantitative studies typically had larger samples that included a maximum of 436 participants [30]. Most participants in the included studies were nurses; only six studies had a sample of doctors or recruited both doctors and nurses. The overall quality of the studies in the sample was high, but several studies shared some limitations, such as small sample sizes (e.g., [31, 32]), incomplete explanations of methodological choices (e.g., [32]), and an insufficient discussion of cultural and religious factors that affect EoLC in Middle Eastern ICUs [33]. Appendix B: Table B1 describes the results of the data extraction process of 27 research studies. Results from quantitative studies are presented in a textual rather than numerical format.

Following the translation of quantitative findings into textual data, descriptive themes were developed, which then were synthesised and we generated five themes: (1) Challenges in EoLC decision‐making in ICUs; (2) Cultural and ethical issues impacting the delivery of EoLC; (3) The need for comprehensive EoLC guidelines; (4) EoLC education and training for ICU clinicians; and (5) holistic support systems for ICU clinicians.

### 4.1 Challenges in EoLC Decision‐Making in ICUs

#### 4.1.1 Communication Challenges

Communication was recognised as an essential but challenging element of EoLC among ICU clinicians in the Middle East [34, 35, 36, 37]. Ineffective communication was reported by nurses and doctors as one of the major challenges they faced when delivering EoLC in ICUs [17, 35, 38, 39]. It was specified that communication barriers existed on all levels: within staff, between staff and patients, and between staff and families. It was found that physicians' and nurses' communication was flawed, which resulted in their poor awareness of patients' needs [36]. For example, nurses felt that they were not asked for their perspective on treatment and were excluded from patient/family meetings. As a consequence of their limited communication with colleagues and families, they perceived that they were offering non‐beneficial treatments and life support that prolonged suffering [40]. Families were often ‘left in the dark’ about the patient's prognosis, as almost half of nurses reported being likely to withhold information about the patient's illness from their families [37].

Communication challenges also negatively affected healthcare providers themselves [38]. Specifically, nurses reported that families often had limited information about patients' conditions and the goals of care. This situation made nurses stressed because the burden of revealing the possibility of the patient's death was left to them, as they were the care providers who spent more time at the bedside than other healthcare professionals [38]. At the same time, nurses in many ME countries are not allowed to explain diagnoses and treatments, which creates professional dilemmas [38, 40]. For some nurses, language barriers were the main communication challenge [31]. The study by O'Neill et al. [41] conducted in Bahrain also revealed that ICU healthcare providers were often not sufficiently fluent in English to communicate with patients and families from different countries. This communication barrier negatively affected their ability to inform families about the patient's state and provide the needed comfort and emotional support.

#### 4.1.2 Relationship‐Building and Lack of Trust

ICU clinicians' interactions with families are a significant source of distress for them as they witness them going through the realisation of patients' imminent death and the subsequent emotional reactions to EoLC [42]. The main problem is that ICU clinicians perceive that many family members are unable to effectively control the complex emotions they experience, such as sadness, anger and anxiety, which complicates their communication with care providers [39, 43]. Clinicians thus feel that the expression of these emotions by family members may cause disruptions in their work and further contribute to already high work‐related stress [33]. It is also often difficult for staff to interact with family members who did not understand what life‐saving measures entailed and had unrealistic expectations about ICU care [38, 40]. Some family members may become aggressive and angry after the topic of EoLC is brought up, while their denial and refusal to accept the possibility of dying create conflicting situations that add to the burden of EoLC provision [38, 44].

Studies exploring health providers' experiences in Middle Eastern ICUs extensively covered family involvement in decision‐making. It was found that the majority of nurses considered family involvement in EoLC essential [43]. Yet, family members were rarely invited to share their opinions on care, which might be due to the limited trust on the part of clinicians who might not see families as useful decision‐making partners [41]. Even though the majority of the physicians agreed that the patient's family should be included in the discussion of EoLC, only a small fraction of them actually engaged them in making DNR‐related decisions [39]. A small number of physicians reported that they almost always/often respected the family's opinion on the limitation of life‐sustaining therapies [39].

#### 4.1.3 Cultural Gender Perceptions and the National Standing of Nurses as Not Being Equal Partners in Care Delivery

Challenges with EoLC also concern the exclusion of nurses from decision‐making. For example, the hospital's decision‐making hierarchy sidelines nurses from crucial care decisions, creating a feeling of helplessness and moral conflict [22]. As a result, nurses may have blamed themselves for failing to provide the EoLC that patients deserved or felt responsible for not delivering the care they thought patients deserved [22]. Similarly, studies conducted in Saudi Arabia and in Egypt found that nurses in ICUs were not treated as knowledgeable independent professionals, so physicians did not consider their opinions when making EoLC‐related decisions [30, 45]. While recognising nurses' limited direct engagement in decision‐making, O'Neill et al. [41] found that they were often engaged indirectly. Physicians tended to ask nurses about the patient's state and family when making EoLC decisions because they perceived them as the most knowledgeable [41].

### 4.2 Cultural and Ethical Issues Impacting the Delivery of EoLC

Cultural and ethical issues were frequently reported within the included studies. The prominence of these issues highlights how deeply rooted cultural beliefs and religious traditions are in EoLC in ICU settings in the Middle East. For example, Benbenishty et al. [35] and O'Neill et al. [41] reported that based on the data collected from nurses, physicians, patients and family members, religious families and patients often requested more extensive treatments despite poor prognoses. Borhani et al. [46] partially explained this phenomenon by stating that some religions, such as Islam, recognise the possibility of miracles, so religious families may view withdrawing life‐sustaining treatments as unacceptable. Alsayed et al. [32] further clarified that most Muslims believe that demonstrating patience at the end of life helps clear their sins. These beliefs directly affect their perceptions of EoLC and explain why patients expect and some ICU clinicians support full intensive medical treatment even when it is futile [33, 47]. However, these beliefs can become a hindrance for some ICU professionals, as shown in several studies [39, 48]. The problem is that they complicate the transition to palliative care, as families cannot accept the need for withholding or withdrawing life‐sustaining treatment and demand treatments that ICU professionals think are unethical to provide. It is not known, though, what role religious leaders may play in EoLC and whether they facilitate decision‐making around EoLC or impede it.

ICU health providers also face ethical and professional dilemmas in relation to DNR orders and the transition to EoLC [49]. Clinicians confided that they frequently found themselves torn between honouring patient autonomy, adhering to religious and cultural expectations, and fulfilling their professional duties [50]. A study involving physicians and nurses in Palestinian ICUs found that the majority felt that religious beliefs greatly affected their perceptions of DNR orders and made it harder for them to deal with DNR‐related issues [50]. These findings underscore the need for additional training and comprehensive and clear guidelines, which are discussed in greater detail in the next sections.

However, research also shows that religion can simplify and enrich EoLC in some cases, allowing ICU clinicians to communicate on difficult topics around death and dying. Specifically, O'Neill et al. [41] argued that religion offers a more expansive vocabulary to talk about EoLC compared to secular language. In turn, it was found that nurses willingly allowed family members to engage in spiritual care and even encouraged such activities as providing an additional source of comfort to both them and patients [34, 46]. Moreover, nurses reported having strong beliefs about the unacceptability of withdrawing life‐sustaining care because they believe in unexpected recovery [46], which in turn reduces decision‐making conflicts since nurses and family members share religious beliefs. Again, the role of religious leaders and the scope of their involvement were not considered in the analysed studies.

### 4.3 The Need for Comprehensive EoLC Guidelines

Healthcare providers' experiences around EoLC in ICUs across Middle Eastern hospitals point to the importance of developing comprehensive EoLC guidelines to guide practice in order to remove uncertainty and reduce moral dilemmas surrounding EoLC [36, 39, 43, 45, 47]. Many hospitals seemed to lack such guidelines at the time when the data were collected, which caused significant confusion for ICU staff [36, 39, 43, 45, 47]. Alshehri et al. [33] revealed a lack of clear guidance on EoLC and variations in palliative care decisions. Their participants disagreed on how DNR orders should be implemented, with or without considering families' wishes. Some physicians argued that this decision should be based on purely medical considerations, whereas others countered that they could not disrespect family wishes [38]. While debates about the legalisation of brainstem death by religious authorities continue, ICU clinicians are left without a clear legal framework for EoLC. As a result, care providers report the dissociation between their attitudes towards EoLC and their practices.

The lack of guidance on EoLC, particularly DNR orders, does not allow nurses to meaningfully engage in decision‐making and further increases the clash between cultural beliefs and professional commitments. For example, it was found that nurses were often reluctant to consider a DNR order because their hospitals had no clear policy in this regard [46]. Another study revealed that nurses in Jordan wanted to be included in DNR decisions but felt that their hospitals did not provide consistent and clear guidance on DNR documentation and decision‐making [43]. In contrast, O'Neill et al. [41] also discovered that nurses believed DNR decisions to be a matter that only God could decide. This discrepancy in findings may be due to the absence of comprehensive and clear guidelines that create a sense of uncertainty and doubt and induce nurses to avoid responsibility for EoLC decisions.

Alanazi's [34] and Alasiry et al.'s [42] findings support the development of EoLC guidelines, showing how these can simplify work for ICU nurses and physicians. Alanazi's [34] study, conducted in Saudi Arabia, showed that comprehensive guidelines outlined how pain management and supportive measures needed to be implemented, which could not only improve patient end‐of‐life experiences but also minimise the risk of unequal treatment [34]. Similar findings were presented by Alasiry et al. [42], who found that hospital guidelines simplified nurses' work and partly decreased physical and emotional stress from caring for dying patients.

### 4.4 EoLC Education and Training for ICU Clinicians

In 14 studies, the need for additional training and awareness raising about EoLC was recognised, [17, 32, 36, 37, 38, 39, 47, 49, 51, 52, 53, 54, 55, 56] which vividly demonstrates the importance of this issue. These interventions are vital because the lack of knowledge and training serves as a major barrier to the effective implementation of palliative care and low acceptance of EoLC [48]. Eltaybani et al. [30] found that ICU nurses and physicians often focused on stabilising patients' conditions and relieving distressing symptoms while overlooking the spiritual and cultural dimensions of EoLC. According to Eltaybani et al. [30] ICU professionals simply considered spiritual elements to be outside of their professional responsibilities. While these findings certainly do not apply to all Middle Eastern ICUs, [34] they demonstrate that some hospitals need to provide additional education and training for their staff to make EoLC more comprehensive and culturally appropriate.

The review found that nurses had significant knowledge gaps regarding palliative care, particularly in the areas of psychosocial and spiritual needs management [31, 45, 52]. In addition, nurses with more than 15 years of work experience were found to have more positive attitudes towards DNR orders [53]. Better training and skills development are essential for modifying nurses' attitudes and equipping them to provide more effective EoLC. It is also recognised that training may help ICU professionals discuss sensitive EoLC issues with families and patients (e.g., DNR orders), improving the quality of communication and decision‐making [50]. According to Almansour et al. [40] communication in ICUs cannot be improved without training team members on how to engage families. Nurses and physicians also need to be guided on how to break bad news to families and how to support them in the most sensitive way to relieve their stress and suffering [46].

### 4.5 Holistic Support Systems for ICU Clinicians Providing EoLC

The final theme concerns the need to develop holistic support systems for ICU clinicians to help them provide better EoLC and experience less stress. One of the elements of these support systems includes support for ICU clinicians (both nurses and doctors) to better attend to their personal and professional needs [34, 44, 50]. For example, it was suggested to provide ICU staff with access to spiritual and psychosocial resources and decrease their responsibilities for some time after a patient's death in order to recover emotionally [33].

Organisational support in the form of clear guidelines and policies (as indicated earlier) is also reported as a key element of support that ICU clinicians need to be able to handle sensitive aspects of EoLC, such as breaking the news to families or discussing treatment withholding/withdrawal [37]. Furthermore, the provision of sufficient human and material resources for EoLC is also vital to support clinicians. To achieve this, organisational modifications are needed. For example, ICUs may need to create interdisciplinary care teams better equipped to meet patients' and families' diverse needs [48]. It may also be used to expand organisational backing through the provision of support from nurse managers, expanding educational opportunities and improving collaboration between ICU staff [30]. Finally, adequate financial and human resource allocation should become central to holistic support systems. Existing research points to the high cost of life‐sustaining treatments, the lack of hospice wards for EoLC patients and staff shortages [22, 36, 46]. Therefore, securing more financial investment in the development of ICUs' capacities and expansion of the staff must be a key priority for hospital management.

## 5 Discussion

Challenges in providing EoLC in ICUs, including communication challenges, problematic interactions with families and decision‐making, were identified in this review as the primary barriers to effective EoLC delivery. Other research from this region and the rest of the world has reflected the salience of the listed problems that healthcare providers face in ICU settings [44, 57, 58]. At the same time, the review revealed cultural and ethical issues unique to countries in the Middle East impacting the delivery of EoLC. These include but are not limited to ambiguous attitudes to DNR orders and the withdrawal/withholding of treatment for patients at the end of life. Limited acceptance of EoLC, which is believed to contradict religious values, has been supported in other studies, validating the conclusions of this review [59, 60]. Interestingly, the findings of this systematic review contrast with the results of prior research conducted in Western countries, indicating that patients' families prefer palliative care over aggressive treatments, perceiving the latter as an indicator of low‐quality care [61, 62]. The significance of these cultural differences across Western and ME countries can hardly be overemphasised. They point to the importance of designing culturally sensitive yet clinically relevant guidelines and standards to guide EoLC care in ME ICUs.

Knowledge gaps among ICU staff were also identified, with prior research confirming that additional on‐site training is needed to make EoLC less stressful for all stakeholders [63, 64, 65]. Existing research underscores the need for cultural training and education for ICU staff to better cater to patients' and families' needs and expectations. Middle Eastern ICUs can learn from best practices in this area, such as programmes like the End‐of‐Life Nursing Education Consortium [66], which offers training in communication, pain management and cultural competency. Nonetheless, it is important to consider cultural differences when translating best practices to Middle Eastern countries. Additional research is needed to better understand the cultural influence on stakeholders' beliefs and attitudes towards EoLC.

The study revealed a demand among ICU nurses and doctors for the introduction of clear EoLC policies and practices in Middle Eastern ICUs. Most of the countries in this region do not have clear national and hospital guidelines on how to communicate with families whose relatives require palliative care. Adopting and adjusting best practices from other countries, such as the Serious Illness Conversation Guide used in the USA and Europe [63], may become a working solution. They can promote clear and empathetic discussions about prognoses and patient preferences. However, in addition to evaluating their potential impact in the Middle Eastern ICUs, policymakers would also need to consider how rigid guidelines might contradict the need for personalised care attuned to patients' cultural and religious values.

Lastly, hospital administration in Middle Eastern ICUs needs to establish holistic support systems for ICU clinicians. It is vital to facilitate the provision of more effective, patient‐centred care while safeguarding staff's emotional health and well‐being. For example, Middle Eastern countries can learn from the example of existing mental health support programmes for healthcare professionals [64] when designing, testing and implementing similar interventions to alleviate the emotional burden on ICU staff and ensure that patients receive compassionate, dignified care. For example, it may be worth considering adopting programmes fostering improvement in ICU clinicians' coping and resilience [65]. Facilitating healthy lifestyles to reduce chronic stress and teaching the staff to utilise cognitive behavioural therapy‐based strategies might also be useful to increase their emotional resilience [65]. Although this review identified infrastructural limitations and a lack of resources as significant problems requiring attention in ICUs, more research is needed to understand how these resource needs vary across different Middle Eastern countries [67].

## 6 Limitations

Some limitations are present in this review. The database searches might not have provided access to all relevant publications, possibly due to the use of keywords that have not accurately captured the focus of the study. Additional studies meeting the inclusion criteria were identified through the manual search of the International Journal of Palliative Care Nursing, as well as the manual screening of the reference lists of relevant articles. The translation of quantitative findings into textual data to make them more homogeneous and suitable for comparison and analysis might have introduced some bias. The shortcomings of retrieving the available literature, as well as potential flawed interpretations due to the methodological heterogeneity of included studies, were minimised by being transparent in describing and justifying every methodological choice and involving two reviewers independently undertaking most parts of the review. Most of the included studies focused on nurses as participants, while the perspectives of physicians were less extensively covered. Since physicians have more decision‐making power in EoLC than nurses, this limitation might have resulted in findings being incomplete. One must also note the lack of consideration in the existing literature of the role of religious leaders in the EoLC in ICUs, which is surprising given the established role of religious beliefs in EoLC‐related decisions. The scope and effect of their engagement in ICUs must be examined in future research to determine how they can be included in holistic EoLC provision that caters to the patients' and families' spiritual needs. Finally, the review included only studies published in English and Arabic. This limitation may have affected the overall findings of the review, potentially leading to some important data being overlooked.

## 7 Implications for Clinical Practice

Existing research underscores the need for cultural training for ICU staff to better cater to patients' and families' expectations. We recommend devising more culturally sensitive, evidence‐based training and education courses about end‐of‐life care aspects, which could be delivered to pre‐registration nurses, doctors and allied health professionals. These courses must recognise the fact that Middle Eastern ICUs employ a large number of non‐ME medical professionals, so they must be educated on the nuances of local cultural and religious perceptions of death and dying. We also advise hospital management in Middle Eastern countries to conduct on‐site courses for ICU staff periodically to ensure they possess up‐to‐date knowledge about best practices in EoLC. To ensure that the patients' and families' voices are considered, we advise assessing the quality of EoLC from their perspective and establishing a convenient feedback collection system that would help hospitals to track progress continuously. Finally, it is worthwhile for the Middle Eastern hospitals' management to adjust their policies regarding ICU staffing and ICU design and design evidence‐based guidelines for the staff to enhance the quality and consistency of end‐of‐life care provision.

## 8 Conclusion

This integrative review synthesised research on how end‐of‐life care is delivered in Middle Eastern ICUs and what challenges clinical professionals face in this regard. It was found that communication barriers often make it difficult for the clinical staff to inform families about EoLC and engage them meaningfully in decision‐making. Unique cultural and religious issues were found to affect the delivery of EoLC, inducing clinical professionals to navigate complex decisions surrounding patient autonomy and dignity. The review exposed the need for comprehensive and clear EoLC guidelines, EoLC education and training for ICU clinicians and holistic support systems are needed to provide more patient‐oriented, meaningful EoLC. More research is needed to explore EoLC from the perspective of a wider range of stakeholders in Middle Eastern countries and determine how best practices can be adjusted to ICUs in this region to enhance EoLC provision.

## Ethics Statement

The authors have nothing to report.

## Consent

The authors have nothing to report.

## Conflicts of Interest

The authors declare no conflicts of interest.

## Data Availability

The data that support the findings of this study are available from the corresponding author upon reasonable request.

## Appendix A

**TABLE A1:** Search terms.

(‘End‐of‐Life Care’ OR ‘Palliative Care’ OR ‘EoLC’ OR ‘End‐of‐Life Issues’ OR ‘Terminal Care’ OR ‘Hospice Care’) **AND** (‘Experiences’ OR ‘Perceptions’ OR ‘Attitudes’ OR ‘Views’ OR ‘Beliefs’ OR ‘Challenges’ OR ‘Barriers’ OR ‘Facilitators’) **AND** (‘Intensive Care’ OR ‘ICU’ OR ‘Critical Care’ OR ‘Critical Care Nurses’ OR ‘ICU Nurses’ OR ‘Healthcare Providers’ OR ‘Nurses’ OR ‘Physicians’ OR ‘Doctors’) **AND** (‘Middle East’ OR ‘Saudi Arabia’ OR ‘Jordan’ OR ‘Egypt’ OR ‘Israel’ OR ‘Iran’ OR ‘Bahrain’ OR ‘Palestine’ OR ‘United Arab Emirates’ OR ‘Kuwait’ OR ‘Oman’ OR ‘Qatar’ OR ‘Lebanon’)

## Appendix B

**TABLE B1:** Summary of findings.

Authors and country	Study design	Study aims	Setting/Participants	Data collection methods	Findings	Quality
Alasiry et al. [42] and Saudi Arabia	Qualitative	Explore nurses' experiences of providing palliative care in ICU settings in Saudi Arabia.	Single site, ICU. Convenience sampling, ICU nurses (*n* = 9).	Semi‐structured interviews. Qualitative content analysis.	**Major themes:** Challenging care in the ICU, collaborative work to meet patients' needs, caring as a holistic approach, experiencing language as a support, experiencing language as a barrier and family‐centred support and care. **Collaboration difficulties**: ICU nurses faced challenges coordinating care among healthcare professionals. **Language barriers**: Communication was a major problem for non‐Arabic‐speaking nurses who provided EoLC in ICUs. **Family‐centred care issues**: Nurses struggled with balancing family involvement and clinical responsibilities.	Good (JBI, 8/10)
Alanazi [34] and Saudi Arabia	Exploratory descriptive & qualitative	Explore ICU nurses' experiences in providing EoLC in Saudi Arabia.	ICU in a tertiary teaching hospital in Riyadh, Saudi Arabia. Purposive sampling, ICU nurses (*n* = 10).	Semi‐structured interviews. Thematic analysis.	**Major themes:** Feeling challenged but motivated, holistic services, collaborative team ethics and caring for the dying and the undying. **Challenges reported:** Stress, feelings of hopelessness, burnout. **Cultural and ethical issues:** Nurses need to provide spiritual support even if they do not share their patients' religion. **Facilitators:** Nurses reported working within the existing EoLC policies that define in detail their practices.	Good (JBI, 8/10)
Almansour et al. [40] and Jordan	Exploratory & descriptive survey	Determine Jordanian critical care staff's perceptions of the intensity and frequency of obstacles and facilitators in providing EOLC.	Two hospitals. Convenience sampling* critical care nurses (*n* = 76) and physicians (*n* = 28).	Adapted National Survey of Critical Care Nurses' Perceptions of End‐of‐life Care questionnaire.	**Barriers**: Family misunderstandings, poor unit design, collaboration difficulties, language barriers, family‐centred care issues faced by ICU nurses, lack of understanding of EoLC principles, insufficient training in palliative care, gaps in healthcare staff knowledge, ineffective communication strategies, environmental challenges and family‐related issues. **Facilitators**: Family acceptance of dying, effective communication.	Good (JBI, 6/8)
Alsayed et al. [32] and Saudi Arabia	Quantitative	Explore the current practice regarding near‐death events in one of the Middle Eastern countries, particularly in Saudi Arabia. Measure the involvement of patients' wishes in EoL medical decisions and analyse the need and usefulness of encouraging writing advance medical directives in this region.	Multiple sites. Convenience Sampling* ICU physicians (*n* = 51).	Questionnaire.	**Advance medical directives**: 31.35% of the physicians witnessed many cases where patients discussed their near‐death wishes with their family members. The majority of physicians (84.31%) believed that these EoL discussions facilitated their work. **Policy issues**: Inconsistencies in policies complicated EoLC practices, contributing to variability in care.	Good (JBI, 7/8)
Alshaikh et al. [31] and Saudi Arabia	Qualitative	Investigate nurses' knowledge about palliative care in an ICU in Saudi Arabia.	Single site, ICU. Convenience sampling, ICU nurses (*n* = 9).	Semi‐structured interviews. Manifest content analysis.	**Unfamiliarity with EoLC concepts**: Barriers included nurses' lack of understanding of EoLC principles. **Lack of education and training**: Insufficient training in palliative care was identified as a significant challenge. **Communication strategies**: Barriers related to ineffective communication strategies were identified, impacting palliative care delivery.	Good (JBI, 8/10)
Alshehri et al. [38] and Saudi Arabia	Qualitative	Explore ICU professionals' perspectives on the provision of palliative and EoLC.	Multiple sites. Purposive sampling* (*n* = 19), physicians (*n* = 12), respiratory therapists (*n* = 4) and dietician (*n* = 1).	Semi‐structured interviews. Interpretive description approach and constant comparative analysis.	**Influence of DNR policies**: EoLC practices were shaped by the presence and application of DNR policies. **Challenges in family involvement**: Engaging families in EoLC decisions presented significant challenges, particularly in terms of communicating care decisions and facing the emotional burden experienced by families. **Policy adherence**: Issues with adhering to existing policies were identified as obstacles to effective care, as decisions were largely driven by care providers' beliefs.	Good (JBI, 8/10)
Attia et al. [33] and Egypt	Cross‐sectional descriptive	Explore critical care nurses' perceptions of barriers and supportive behaviours in providing EoL care to patients and their families.	Multiple sites. Convenience sampling, nurses (*n* = 70)	Structured interview sheet adapted from Beckstrand and Kirchhoff.	**Barriers to palliative care**: Environmental challenges (e.g., heavy workload and poor ICU design), family‐related issues (families not understanding the meaning of EoLC) and knowledge gaps (e.g., family grieving) were identified as key barriers. **Supportive staff behaviours**: Supportive behaviours (e.g., communication, emotional support after patient's death) from staff were observed despite the presence of these barriers.	Good (JBI, 7/8)
Azab et al. [39] and Egypt	Survey‐based observational study	Explore physicians' attitudes towards EoLC and the reported practice in adult ICUs.	Multiple sites. Random sampling, physicians (*n* = 100).	Structured questionnaire developed specifically for the study.	**Support for DNR orders**: Doctors generally supported DNR orders but expressed concerns about legal and ethical implications of limiting EoLC. **Disconnect between theory and practice**: The study highlighted a disconnect between theoretical support for DNR orders and their actual implementation, suggesting a need for clearer guidelines and training.	Fair (JBI, 5/8)
Basal et al. [47] and Egypt	Descriptive mixed methods	Examine critical care nurses' knowledge, practice, barriers and facilitators towards palliative care for critically ill patients.	Single site, ICU and Oncology ICU. Convenience sampling, nurses (*n* = 70).	Structured interview, observational checklist, perception questionnaire (specifically developed for the study).	**Gaps in knowledge and practice**: Nurses demonstrated significant gaps in knowledge and practice related to EoLC. **Barriers:** Not enough time to provide quality EoLC. **Variability in care quality**: Variability in the quality of care was noted, highlighting the need for improved education and consistent practices to enhance EoLC outcomes.	Good (JBI, 7/8)
Benbenishty et al. [35] and Israel	Prospective qualitative	Explore European and Middle Eastern ICU nursing ceremonies and rituals surrounding post‐death care.	Multiple sites. Purposive sampling*, nurses (*n* = 23).	Face‐to‐face interviews. Thematic analysis.	**Cultural practices and emotional support**: Cultural practices, rituals and family contact were reported as playing significant roles in EoLC. **Cultural sensitivity**: The need to integrate cultural sensitivity into EoLC practices to better support patients and families.	Good (JBI, 8/10)
Borhani et al. [46] and Iran	Qualitative	Investigate ICU nurses' perspectives of EoLC in the southeast of Iran.	Multiple sites. Purposeful sampling, nurses (*n* = 12).	Semi‐structured interviews. Inductive coding.	**Major themes**: Commitment to care, awareness of dying patients, caring relationships and dealing with challenges and ethical issues. **Ethical dilemmas and cultural challenges**: Nurses reported facing ethical dilemmas and cultural challenges in EoLC, particularly in balancing empathy with ethical responsibilities. **Challenges in care**: Nurses identified difficulties in managing these ethical and cultural issues in their day‐to‐day practice.	Good (JBI, 7/10)
Eltaybani et al. [30] and Egypt	Cross‐sectional survey	Assess palliative care education, practice and perceived competence among adult ICU nurses. To explore factors moderating palliative care nursing practice and perceived competence. To evaluate the construct and criterion validities of the PEOL Care Index.	Multiple sites. Random sampling, nurse managers (*n* = 33) and staff nurses (*n* = 403).	Self‐administered questionnaires.	**Barriers**: Insufficient palliative care education and inconsistent care practices were reported as barriers. **Determinants of palliative care**: In‐service training on palliative care EoLC, higher job satisfaction and higher organisational support. **Improvement suggestions**: The study suggested enhancing education and developing comprehensive EoLC policies and practices to improve palliative care delivery.	Good (JBI, 7/8)
Ganz and Sapir [36] and Israel	Descriptive, correlational& cross‐sectional design	Describe ICU nurses' perceptions of quality palliative EoLC, barrier intensity and frequency to palliative care and the relationship between them.	Two hospitals in Israel. Convenience sample (*n* = 126).	Questionnaires: a personal characteristics questionnaire, the Quality of Palliative Care in the ICU and a revised Survey of Oncology Nurses' Perceptions of End‐of‐Life Care.	**Main findings**: The quality of palliative care was moderate despite moderate barriers (e.g., perceived lack of education). **Barriers:** Interaction with patients and families, communication within the team, environmental factors (unit structure). **Impact of education**: Education in palliative care significantly improved knowledge and attitudes, highlighting the need for targeted training.	Good (JBI, 7/8)
Iranmanesh et al. [51] and South‐East Iran	Descriptive correlational	Determine the correlation between the professional autonomy of oncology and ICU nurses and their attitudes towards the care of dying patients.	Multiple sites. Convenience sampling, oncology and ICU nurses (*n* = 155).	Questionnaires: Frommelt Attitude Towards Care of the Dying (FATCOD) and Pankratz Nursing Questionnaire (PNQ).	**Attitudes towards EoLC**: Nurses generally held neutral to moderately positive attitudes towards EoLC. **Influence of professional autonomy and education**: Positive correlations were observed between attitudes towards EoLC and factors such as professional autonomy and education. **Impact of education and experience**: The study highlighted the impact of education and experience on shaping attitudes towards EoLC.	Good (JBI, 6/8)
Khalaileh et al. [43] and Jordan	Cross‐sectional survey	Examine ICU nurses' attitudes towards and experiences of DNR decisions.	Multiple sites. Self‐selecting sample, nurses (*n* = 111).	Perceptions and Attitudes to DNR questionnaire developed by Thibault‐Prevost et al. [68]	**Support for physician and family involvement**: Nurses supported physician and family involvement in DNR decisions. **Limited engagement**: Nurses had limited practical engagement in DNR discussions. **Suggested improvements**: The study suggested that more comprehensive and unambiguous policies and increased nurse involvement could improve DNR practices.	Fair (JBI, 5/8)
Khater et al. [48] and Jordan	Qualitative	Explore the perceived barriers to implementing palliative care in ICUs.	Two ICUs. Purposive sampling* clinicians (*n* = 17).	Semi‐structured interviews. Thematic analysis.	**Main themes**: ICU as a demanding and complex clinical setting; lack of preparation to provide palliative care; palliative care as a nicety, not a necessity; healthcare system‐related barriers; and lack of cultural acceptance of palliative care. **Need for improvement**: The study highlighted the need for improved training and cultural adaptation to enhance palliative care practices.	Good (JBI, 8/10)
Mani et al. [45] and Saudi Arabia	Mixed‐method sequential explanatory	Explore nurses' perceptions of obstacles to the EoLC provision in ICUs.	Single site. Convenience sampling, nurses (*n* = 87).	Modified version of a questionnaire developed by Beckstrand and Kirchhoff.	**Obstacles to EoLC**: Major obstacles included concerns regarding patient's family, varied opinions among physicians, cultural differences and language barriers. **Need for education and awareness**: The study emphasised the need for increased education and awareness to address these barriers and improve EoLC practices.	Good (MMAT, 7/10)
Mrayyan et al. [52] and Jordan	Cross‐sectional & comparative study	Examine Jordanian nurses' perceptions of the obstacles and supportive behaviours of EoLC in ICUs and identify the differences based on the participants' demographics.	Multiple sites. Convenience sampling, nurses (*n* = 230).	Questionnaire.	**Obstacles to EoLC:** Lack of nursing education and training. **Facilitators:** Family members' acceptance that the patients are dying when nurses offer them their support. Nurses' EoLC experiences differed depending on certification, ICU type, facility type, number of beds and the number of hours worked per week.	Good (JBI), 8/10
Naghshbandi et al. [53] and Iran	Descriptive‐analytical (quantitative)	Explore ICU nurses' attitudes towards DNR orders.	Multiple sites. Random sampling, nurses (*n* = 255).	Questionnaire (demographic information and 11 questions with a 5‐point Likert scale about the attitude towards the DNR order).	**Attitudes towards DNR orders**: Negative attitudes towards DNR orders were found among nurses. **Impact of experience**: Nurses with more experience had more positive attitudes towards DNR orders. **Influence of education**: The findings suggest that experience and education impact attitudes towards DNR decisions.	Fair (JBI, 5/8)
O'Neill et al. [41] and Bahrain	Qualitative	Explore nurses' care practices at EoL. To describe and identify EoLC practices that nurses contribute to, with an emphasis on culture, religion and professional identity.	Two ICUs. Theoretical sampling (*n* = 10).	Semi‐structured interviews, grounded theory.	**Avoidance of death discussions**: The study revealed a tendency among healthcare professionals to avoid discussions about death. **Signalling death to families**: This avoidance led to a process of signalling death to families and shifting care responsibilities to families. **Need for direct communication**: The study highlighted a need for more direct communication and support strategies in EoLC.	Good (JBI, 9/10)
Rawas et al. [22] and Saudi Arabia	Qualitative	Investigate healthcare professionals' moral distress and ethical decision‐making.	Single site. Purposive sampling, nurses (*n* = 5).	Semi‐structured interviews. Content analysis.	**Moral distress and ethical dilemmas**: Nurses experienced moral distress and ethical dilemmas related to workload and hierarchical structures. **Feelings of guilt and constraint**: Nurses reported feelings of guilt and constraint. **Need for ethical support**: The study emphasised the need for addressing these issues through ethical support and improved practice environments.	Good (JBI, 6/8)
Razban et al. [37] and Iran	Cross‐sectional descriptive	Explore oncology and ICU nurses' attitudes towards palliative care.	Multiple sites. Convenience sampling* oncology and ICU nurses (*n* = 140).	Questionnaire designed specifically for the study.	**Attitudes towards palliative care**: Nurses exhibited moderately negative to neutral attitudes towards palliative care. **Demographic correlations**: Significant correlations with demographic factors such as education and experience were noted. **Suggested interventions**: The findings suggest that targeted interventions could improve attitudes and care practices.	Good (JBI, 4/5)
Saifan et al. [50] and Palestine	Descriptive correlational cross‐sectional	Determine whether Palestinian physicians and nurses agree with legalising the DNR order and if their religion, culture or both influence their attitudes.	Multiple sites. Convenience sampling, physicians (*n* = 48) and nurses (*n* = 45).	Questionnaire.	**Support for legalising DNR orders**: Strong support for legalising DNR orders was found among both doctors and nurses. **Influence of religious and cultural factors**: Religious beliefs and cultural factors significantly influenced attitudes towards DNR orders. **Role of cultural and religious contexts**: The findings suggest that cultural and religious contexts play a significant role in shaping attitudes towards DNR orders.	Good (JBI, 6/8)
Scheinberg‐Andrews and Ganz [54] and Israel	Cross‐sectional	Describe and compare self‐perceived EoL knowledge, attitudes, behaviours and practices of ICU nurses compared to oncology nurses.	Multiple sites. Random Sampling*, oncology nurses (*n* = 79) and ICU nurses (*n* = 47).	A 28‐item EOLC‐ICU questionnaire based on that created by Montagnini et al.	**Experience differences**: ICU nurses had significantly more experience compared to oncology nurses. **Differences in knowledge, attitudes and behaviours**: Differences in knowledge, attitudes and behaviours towards palliative care were observed between ICU and oncology nurses. **Impact of education and experience**: Education and personal experience positively impacted knowledge, attitudes and behaviours, revealing gaps that need to be addressed for improved EoLC practices.	Good (JBI, 7/8)
Subih et al. [55] and Jordan	Cross‐sectional& descriptive correlational	Evaluate the levels of nurses' knowledge about EOLC, explore the relationships between EoLC knowledge and demographic variables, and determine predictors of EoLC knowledge.	Multiple sites. Convenience sampling, nurses (*n* = 175).	The End‐of Life Professional Caregiver Survey (EPCS).	**Knowledge of EoLC**: Nurses exhibited moderate to high levels of knowledge about EoLC. **Significant predictors**: Age, work experience and specific training were significant predictors of EoLC knowledge. **Impact of training**: The study highlighted the positive impact of palliative care training on nurses' knowledge of EoLC.	Good (JBI, 7/8)
Tirgari et al. [49] and Iran	A cross‐sectional descriptive	Assess difficulties in providing EoLC among ICU nurses.	Multiple sites. Convenience sampling* (*n* = 123).	Difficulties felt by intensive care unit (ICU) nurses providing end‐of‐life care (DFINE) questionnaire.	**Difficulties in transitioning to EoLC**: ICU nurses reported moderate difficulties in transitioning to EoLC, particularly in managing life‐sustaining treatments and meeting staffing needs. **Experience and challenges**: Increased experience was correlated with greater challenges, suggesting a need for improved support systems and education.	Good (JBI, 7/8)
Valiee et al. [56] and Iran	Qualitative exploratory	Examine ICU nurses' experiences in providing EoLC.	Single site. Purposive sampling, nurses (*n* = 10).	Semi‐structured interviews. Content analysis.	**Main themes**: Emotional burden and values and beliefs. **Emotional distress**: Nurses experienced significant psychological pressure, including feelings of hopelessness and depression. **Challenges with family reactions**: Nurses struggled with managing family reactions during EoLC. **Influence of personal beliefs and ethical responsibilities**: The study highlighted the influence of personal beliefs and ethical responsibilities on nurses' approaches to EoLC.	Good (JBI, 7/10)

* Not reported by the author, assumption of the review team.
